# Roles of citrus fruits on energy expenditure, body weight management, and metabolic biomarkers: a comprehensive review

**DOI:** 10.1093/nutrit/nuad116

**Published:** 2023-09-13

**Authors:** Merve Nur Aslan, Betül Sukan-Karaçağıl, Nilüfer Acar-Tek

**Affiliations:** Faculty of Health Sciences, Department of Nutrition and Dietetics, Bolu Abant Izzet Baysal University, Bolu, Turkey; Department of Nutrition and Dietetics, Institute of Health Sciences, Gazi University, Ankara, Turkey; Department of Nutrition and Dietetics, Institute of Health Sciences, Gazi University, Ankara, Turkey; Faculty of Health Sciences, Department of Nutrition and Dietetics, Gazi University, Ankara, Turkey

**Keywords:** adipogenesis, citrus, energy metabolism, flavonoids, lipid metabolism, thermogenesis

## Abstract

Citrus fruits are widely consumed for their nutritional and health benefits. They belong to the Rutaceae and have many varieties, such as sweet orange (Citrus sinensis), which is the most popular. Citrus fruits are rich in water (>80%), dietary fiber, and vitamins. They also contain bioactive components, which may modulate energy metabolism and lipid oxidation through various mechanisms. These mechanisms include stimulating β3-adrenergic receptors, increasing mitochondrial biogenesis and thermogenesis, activating AMP kinase and peroxisome proliferator-activated receptor-gamma coactivator-1α pathways, inhibiting lipogenesis and lipid accumulation, and inducing browning of white adipose tissue. This review summarizes the mechanisms and outcomes of citrus fruits and their metabolites on energy metabolism and body weight in different experimental models. The literature was searched for in vitro and in vivo animal and human studies that investigated the effects of citrus consumption on energy expenditure, thermogenesis, adipogenesis, and lipid accumulation. Citrus fruits and their metabolites have shown promising effects on energy metabolism and lipid oxidation in in vitro and in vivo animal studies. However, the evidence from human studies is limited and inconsistent. Possible reasons for the discrepancy are briefly discussed, and knowledge gaps and research needs are identified for future studies. Citrus fruits may have beneficial effects on energy metabolism and body weight, but more rigorous and well-designed human trials are needed to confirm their efficacy and safety.

## INTRODUCTION

Citrus fruits, which are produced in almost all countries of the world, have an important place in the human diet in their fresh or processed forms. Although China, Brazil, India, Mexico, and the United States share the lead in the amount of citrus production in 2020, according to the report of the Food and Agriculture Organization, citrus is produced in >140 countries.^1^ Sweet orange (*Citrus sinensis*) constitutes more than half of citrus production. Citrus fruits are commonly consumed fresh, with about one-third used after processing.^2^ Orange juices constitute 85% of processed products.^3^ Classification of citrus fruits belonging to the Rutaceae is quite complex, and recent studies have adopted 4 basic classifications: *C. maxima, C. medica, C. reticulata, and C. micrantha*). However, there are still contradictions about the origin of certain varieties.^4^^,^^5^ The botanical names of edible citrus and their commonly used names are given in Table 1.^5^

**Table 1:** **Common and botanical names of citrus** ^5^

Botanical name	Common name
*Citrus sinensis*	Sweet orange
*C. reticulata*	Mandarin (tangerine)
*C. grandis/maxima*	Pummelo (pamplemousse)
*C. medica*	Citron
*C. mitis*	Calamondin
*Fortunella margarita*	Kumquat
*C. aurantium*	Sour or bitter orange
*C. paradisi*	Grapefruit
*C. bergamia*	Bergamot
*C. aurantifolia*	Lime
*Poncirus trifoliata*	Trifoliate orange

Citrus fruits with >80% water content are rich in simple sugars and dietary fiber. The energy source of citrus, which has a very low protein and fat content, is carbohydrates.^3^^,^^6^ Citrus fruits are rich in micronutrients, including ascorbic acid, and are also a source of vitamins such as thiamine, pyridoxal phosphate, and folate. Additionally, they contain key minerals like potassium.^7^ In addition to all these primary metabolites, the secondary metabolites in citrus fruits have attracted attention in recent years.^6^ Among these are many bioactive components such as flavonoids (eg, naringenin, hesperidin), alkaloids (eg, synephrine), terpenes (eg, limonene), and carotenoids (eg, β-cryptoxanthin).^8^^,^^9^ These bioactive metabolites have antioxidant,^10^ antidiabetic,^11^ cardioprotective,^12^ anticancer, and anti-inflammatory properties.^13^ Citrus and its metabolites have been discussed in various aspects in terms of health in in vitro,^14^^,^^15^ in silico,^16^ and in vivo animal^17^ and human^18^ studies.

The effect of citrus fruits on body weight is 1 of the issues that has attracted attention in the past 20 years. Phytochemicals, especially, have attracted attention in the scientific world.^15^^,^^19^^,^^20^ Citrus flavonoids have been associated with reducing adiposity and regulating obesity-related enzymes such as carnitine palmitoyltransferase-I and stearoyl-coenzyme A desaturase, among others.^21^^,^^22^ In a meta-analysis, consumption of citrus and its extracts were reported to decrease body mass index (BMI), waist and hip circumference, and body weight, and these effects were more pronounced at higher doses.^23^ Wang et al^24^ found that orange juice consumption by adults was associated with lower BMI, waist circumference, and body fat percentage. It has been reported that bitter orange (*C. aurantium*) extract and its primary protoalkaloidal component, *p*-synephrine, have thermogenic and lipolytic activities, affect energy metabolism, and are effective in weight management.^25^ Additionally, there is scientific evidence that *p*-synephrine does not have any side effects in acute or long-term use.^26^ In this review, we investigated the effectiveness of bioactive components of citrus fruits on energy expenditure, weight management, and metabolic effects as reported in in vitro and in vivo studies.

## LITERATURE REVIEW

In this study, PubMed (used to search MEDLINE), Web of Science, Cochrane Central Register of Controlled Trials, SPORTDiscus, and Scopus databases, and the Google Scholar website for gray literature, were searched without time limitation. During the screening, the botanical and common names of citrus given in Table 1,^5^ citrus metabolites (eg, vitamin C, dietary fiber, naringenin, synephrine) were used. Additionally, the keywords “resting metabolic rate,” “thermogenesis,” “energy metabolism,” “energy expenditure,” and “weight loss” were used to explore the role of citrus in energy metabolism and the outputs of the studies. The primary and secondary metabolites included in the review were determined assuming that citruses could be the main sources in the diet. Table 2^27–59^ summarizes the English-language, full-text articles about the role of citrus and its metabolites in energy metabolism that were included in the study.

**Table 2:** Summary of clinical studies with citrus fruits

Reference	Type of study	Type of citrus	Doses and duration	Participants	Key results
Kegele et al (2019)^27^	RCT	*C. sinensis*	First group (n = 35): 0.5 g of extract Second group (n = 35): 1 g of extract Third group (n = 36): placebo for 3 mo	Male and female adults with abdominal fat >20% of body weight	Muscle mass increased by 0.58% in the 0.5 g group and 7.81% in the 1 g group. Fat mass decreased by 0.64% in the 0.5 g group and by 11.89% in the 1 g group.
Li et al (2020)^28^	RCT	*C. sinensis*	First group (n = 8): 200 mL of red orange juice Second group (n = 8): placebo twice/d for 2 wk	n = 16 healthy men and premenopausal women aged 20–45 y with BMI >25 kg/m^2^	There was no significant change in HDL, LDL, TG, TC, and BP levels compared with baseline.
Cardile et al (2015)^29^	RCT	*C. sinensis*	First group (n = 30): Moro orange juice extract Second group (n = 30): nothing for 12 wk	n = 60 healthy people with a BMI between 25 and 35 kg/m^2^	Body weight*, BMI*, WC*, and HC* values decreased in the supplement group. No change was found in the control group.
Niv et al (2012)^30^	RCT	*C. sinensis*	First group (n = 29): 500 mL of fresh pasteurized orange juice Second group (n = 19): 500 mL levan-containing orange juice per day for 2 mo	n = 48 healthy people (aged 18–60 y)	There was no significant change in body weight, HDL, LDL, TG, TC, and BP values in both groups.
Azzini et al (2017)^31^	Cross-sectional Study	*C. sinensis*	Volunteers were given red orange juice 500 mL/d for 12 wk.	n = 20 women with a mean age of 36 ± (standard deviation) 7 y and a mean BMI of 34.4 ± 4.8 kg/m^2^.	There was no significant change in body weight, HDL, LDL, TG, TC, BP, WC, and HC values at the end of 12 wk.
Briskey et al (2022)^32^	RCT	*C. sinensis*	First group (n = 90): diet + 400 mg/d moro orange juice extract capsule + exercise Second group (n = 90): diet + exercise for 6 mo	A total of 180 overweight (25 < BMI < 35 kg/m^2^) healthy men and women aged 20–65 y	There was a significant decrease in body weight*, BMI*, WC*, and HC* in both groups, and this decrease was higher in the extract group. There was no significant change in HDL, LDL, or TC levels.
Simpson et al (2012)^33^	RCT	*C. sinensis*	First group (n = 16): 250 mL orange juice Second group (n = 16): 250 mL orange-flavored beverage per day for 12 wk	n = 32 overweight women aged 20–45 y with HOMA-IR >1.5 and a BMI of 27–35 kg/m^2^	There was no significant change in LDL, HDL, TC, SBP, DBP, apo-A1, and apo-B levels; HOMA-IR; and body weight of the groups at the end of 12 wk.
Basile et al (2010)^34^	Cross-Sectional Study	*C. sinensis*	For 8 wk, men were given 750 mL/d orange juice, and women were given 500 mL/d orange juice.	n = 21 healthy women aged 20–53 y and 20 healthy men aged 21–44 y	There was no significant change in orange juice consumption, body weight, and BMI in either sex. Although WC* decreased in women, no change was found in men. TC* and LDL* levels were decreased in both sexes. HDL* increased in women, whereas TG* and DBP* levels decreased significantly in men.
Cesar et al (2010)^35^	Cohort Study	*C. sinensis*	First group: normocholesterolemic (n = 31) Hypercholesterolemic (n = 14): 750 mL of orange juice Second group (n = 8): nothing daily for 60 d	n = 53 male and female adults aged 36–44 y	No significant changes were found in the groups in BMI, WC, and HDL levels. TC** and LDL** levels were decreased in the hypercholesterolemic group. TG* level increased in the control group.
Asgary et al (2014)^36^	RCT	*C. sinensis*	First group (n = 11): 500 mL commercial orange juice Second group (n = 11): 500 mL fresh orange juice twice daily for 4 wk	n = 22 healthy volunteers (7 men and 15 women) aged 18–59 y	No significant change was found in the weight, BMI, WC, apo-B, HDL, TC, and TG levels of both groups. A significant decrease in LDL* level was found in the fresh orange juice group.
Pittaluga et al (2013)^37^	Cohort Study	*C. sinensis*	First group (n = 15): 250 mL of fresh red orange juice Second group (n = 7): nothing 3 times/d 1 h before each meal for 4 wk	n = 22 healthy individuals who exercised regularly for 6 mo and were fed a Mediterranean diet	There was no significant change in BMI, body fat mass, and heart rate in both groups.
Büsing et al (2019)^38^	Cohort study-cross-over	*C. sinensis*	First group (n = 12): orange juice (43 kcal/100 mL and 8.8 g sugar/100 mL) Second group (n = 14): cola drink (44 kcal/100 mL and 11.1 g sugar/100 mL) 3 times/d for 2 wk	n = 26 healthy adults	There was no significant change in body weight between groups.
Hägele et al (2018)^39^	Cohort Study-Cross-over	*C. sinensis*	Orange juice (43 kcal/100 mL and 8.9 g sugar/100 mL) in an amount to meet 20% of the daily energy needs 3 times/d for 2 wk was given to the first group with meals and the second group between meals. After 1 wk of washout, the application was repeated by crossing.	n = 26 healthy adults (13 women, 13 men) aged 20–45 y	There was no significant change in body weight, HOMA-IR, and TG groups and between groups. Consumption of orange juice with meals decreased body fat mass*, and consumption between meals increased body fat mass**. Fat mass** change was different between groups.
Stookey et al (2012)^40^	RCT- crossover	*C. sinensis*	On 2 separate days, the groups were crossed and individuals consumed 500 mL of water or orange juice with breakfast.	Aged 11–17 y with a BMI <85th percentile. n = 7 adolescents and 10 adults (19–38 y) with a BMI 18.5–24.9 kg/m^2^	At 180 min after breakfast, the plasma insulin to glucose ratio* increased in adolescents and adults compared with those drinking orange juice. 30 min after breakfast, fat oxidation* decreased compared with those drinking water at breakfast with an orange drink in both age groups. But NPREE and NPRER did not change.
Sakaki et al (2021)^41^	Cohort Study	*C. sinensis*	The survey data were followed from 2004 to 2008 with an interval of 2 y.	n = 7301 children aged 9–16 y were included in the study.	Orange juice consumption was not associated with BMI and weight.
Aptekmann et al (2010)^42^	Cohort Study	*C. sinensis*	First group (n = 13): 500 mL of orange juice Second group (n = 13): nothing daily for 3 mo	n = 30 physically active premenopausal women aged 30–48 y	Weight*, BMI*, SFT* (triceps, thigh), lactate, TC*, and LDL* levels decreased in the experimental group compared with the control group; HDL*, TG*, and SFT* values (biceps) increased.
Simpson et al (2016)^43^	RCT	*C. sinensis*	First group (n = 18): 250 mL of orange juice Second group (n = 18): orange-flavored beverage for 12 wk	n = 36 healthy men aged 40–60 y with BMI 27–35 kg/m^2^	BMI, weight, WC, HC, WHR, HOMA-IR, % body fat, TC, HDL, LDL, TG, apo-A1, and apo-B values did not change significantly between groups.
Ribeiro et al (2017)^44^	RCT	*C. sinensis*	First group (n = 39): 500 mL of orange juice and a low-calorie diet (500 kcal reduced) Second group (n = 39): only a low-calorie diet for 12 wk	n = 78 individuals aged 18–50 y with BMI: 30–40 kg/m^2^	Weight, BMI, WC, HC, WHR, body fat, FG, HDL, TG, AST, and ALT values did not change. At wk 8 and 12, insulin* and HOMA-IR* values were decreased in the experimental group compared with the control group. At wk 12, LDL* level decreased.
O’Neil et al (2011)^45^	Cross-sectional study	*C. sinensis*	Data were obtained through surveys (NHANES) collected annually between 2003 and 2006. First group (n = 2183): 100% orange juice consumer Second group (n = 5067): nonconsumer	n = 7250 individuals between the ages of 2 and 18 y were included.	The group that consumed orange juice had lower levels of WC* and LDL* compared with the group that did not consume orange juice. Weight, BMI, SBP, DBP, TC, TG, HDL, apo-B, and insulin levels did not differ between groups.
Rumbold et al (2015)^46^	RCT-crossover	*C. sinensis*	Participants were given 600 mL of skim milk or 600 mL (475 mL of orange juice and 125 mL of water) of orange juice after 30 min of cycling exercise.	Nine recreationally active women aged between 18 and 21 y and BMI of 19–25 kg/m^2^ were included.	Vo^2^_peak_ was not different between groups. Relative energy intake* at the test meal decreased in milk consumption.
Fidélix et al (2020)^47^	Cohort Study	*C. sinensis*	Participants followed a regular diet with no orange juice for 30 d, 300 mL of orange juice/d for 60 d, and no orange juice again for 30 d.	n = 10 healthy women aged between 20 and 35 y	BMI, weight, and body fat did not change over the 120-d period. During the drinking period of orange juice, FG*, insulin*, HOMA-IR*, TG*, TC*, and LDL* values decreased.
Rangel-Huerta et al (2015)^48^	RCT	*C. sinensis*	First group (n = 100): 500 mL high-polyphenol orange juice Second group (n : 100): 500 mL of normal polyphenol orange juice for 12 wk	n = 100 individuals with a BMI 25–40 kg/m^2^ or a WC >94 cm in men and >80 cm women	Weight*, BMI,* WC*, and FPS* increased in both groups. SBP*, DBP*, insulin*, TG*, and apo-B* levels were decreased in the second group. Apo-A1* was decreased in the first group. HOMA-IR, TC, HDL, and LDL levels were unchanged.
Aptekmann et al (2013)^49^	Cross-sectional Study	*C. sinensis*	The data were created by analyzing data from those who drank ≥1 glass of orange juice/d and those who did not drink it at all in the last year. Consumers (n = 50); nonconsumers (n = 79)	n = 129 individuals aged 18–66 y	There was no significant difference in TG, HDL, apo-A1, BMI, weight, body fat, and WC values. Normolipidemic or hypercholesterolemic individuals who consumed orange juice had less TC*, LDL*, and apo-B* compared with those who did not.
Dallas et al (2008)^50^	RCT	*C. sinensis* *C. aurantium* *C. paradisi*	First group (n = 10): 4 capsules containing citrus mix extract Second group (n = 10): 4 capsules containing placebo for 12 wk	n = 20 healthy individuals aged 25–55 y with BMI 27–33 kg/m^2^	At the end of wk 4 and 12, the experimental group's BMI*, weight*, and body fat* values had decreased compared with the placebo.
Silveira et al (2015)^51^	Cross-sectional Study	*C. sinensis*	First group (n = 17): normal weight and 750 mL of orange juice Second group (n = 12/6): overweight/obese group and 750 mL of orange juice daily for 8 wk	n = 35 healthy and nonsmoking individuals aged 18–29 y	In the first group, after 8 wk, TC*, HDL*, LDL*, apo-A1*, FG*, HOMA-IR*, and SBP* decreased; weight, BMI, body fat, WC, apo-B, TG, and DBP values did not change. In the second group, TC, LDL, and DBP values decreased after 8 wk, whereas other values remained unchanged.
Penzak et al (2001)^52^	Cross-sectional Cross-over	*C. aurantium*	Participants drank 8 oz (237 mL) of bitter orange juice (phase 1) or water (phase 2) 8 h before arriving at the study unit. After coming to the research unit, their measurements were taken, and 8 oz (237 mL) of bitter orange juice (phase 1) or water (phase 2) was given again. Measurements were repeated hourly for 5 h.	n = 12 healthy individuals aged 20–27 y	The bitter orange juice had no significant effects on SBP, DBP, or arterial pressure values, and heart rate compared with water.
Silver et al (2011)^53^	RCT	*C. paradisi*	First group (n = 29): half a fresh grapefruit Second group (n = 28): grapefruit juice (127 g) Third group (n = 28): water (127 g) for 12 wk	n = 85 adults aged 21–50 y with BMI of 30–39.9 kg/m^2^	BMI*, WC*, and body fat percentage* decreased in all groups at the end of 12 wk. There was no significant difference in REE, RQ, BMI, WC, HDL, TC, TG, SBP, or DBP values within and between groups.
Fujioka et al (2006)^54^	RCT	*C. paradisi*	First group (n = 24): grapefruit capsule + 207 mL of apple juice (capsule) Second group (n = 21): placebo capsule + 237 mL grapefruit juice Third group (n = 24): half a fresh grapefruit + placebo capsule Fourth group (n = 22): placebo capsule + 207 mL of apple juice 3 times/d for 12 wk	n = 91 obese adult men and women with BMI = 30–40 kg/m^2^	The fresh grapefruit group had greater weight loss* than the placebo group. HDL, TG, WC, and BP values did not change significantly.
Dow et al (2012)^55^	RCT	*C. paradisi*	First group (n = 39): half a peeled grapefruit Second group (n: 32): nothing 3 times/d for 6 wk	n = 85 premenopausal healthy individuals with BMI ≥ 25–45 kg/m^2^	WC***, WHR*, SBP*, TC*, and LDL*** values decreased in the experimental group after the intervention. BMI, WC, weight, HC, WHR, SBP, DBP, heart rate, TC, TG, HDL, and LDL did not change between the groups after the intervention.
Taghizadeh et al (2016)^56^	RCT	*C. aurantifolia*	First group (n = 24): 75 mg of *Cumin cyminum* L. (cumin) and lime capsule Second group (n = 24): 25 *C. cyminum* L. (cumin) and lime capsule Third group (n = 24): placebo twice daily for 8 wk	n = 72 healthy aged 18–50 y with BMI ≥ 25 kg/m^2^	In both experimental groups, weight***, BMI***, and FG*** values decreased compared with the placebo group. In addition, the high-dose group LDL* TC**, and TG* levels decreased compared with the low-dose and placebo groups. HOMA-IR, HOMA-β, and HDL values remained unchanged.
Hashemipour et al (2016)^57^	RCT	*C. aurantifolia*	First group (n = 30): lemon peel–powder capsule Second group (n = 29): placebo for 4 wk	n = 60 healthy individuals aged 10–18 y with BMI > 85th percentile	WC, NC, BMI, DBP, SBP, FG, TC, and LDL values did not change between groups after the intervention. HDL** increased in the placebo group. BMI***, SBP**, TC*, and LDL* levels decreased before the intervention in the experimental group after the intervention. BMI*** was also decreased in the placebo group.
Ferro et al (2020)^58^	RCT	*C. bergamia*	First group (n = 51): *C. bergamia* extract + diet (<500 kcal) Second group (n = 51): placebo + diet (<500 kcal)	n = 102 individuals aged 30–75 y	BMI**, weight,** and controlled attenuation parameter score* decreased compared with the control group. WC, HC, TG, HDL, LDL, TC, FG, AST, and ALT values were unchanged. In subgroup analyses, men*, those older than 50 y**, those with android obesity*, and those with obese/overweight** had decreased weight in the experimental group compared with the control.
Lin et al (2022)^59^	RCT	*C. reticulata*	First group (n = 10): *C. reticulata* drink (50 mL and with 20% *C. reticulata* extract) Second group (n = 10): placebo drink (50 mL) twice a day for 6 wk	n = 20 healthy individuals (aged 18–70 y) with BMI ≥ 24 kg/m^2^ or body fat > 30%	At the end of 4, 6, and 8 wk, body weight* decreased in the experimental group. In addition, WC* decreased at the end of the 6th wk and AST* level decreased at the end of the 8th wk. Body fat, DBP, ALT, TC, TG, FG, and BMI levels did not change.

* *P* < 0.05,

** *P* < 0.01,

*** *P* < 0.001,

**** *P* < 0.0001.

*Abbreviations:* ALT, alanine transaminase; apo-A1, apolipoprotein A1; apo-B, apolipoprotein B; AST, aspartate aminotransferase; BMI, body mass index; BP, blood pressure; DBP, diastolic blood pressure; FG, fasting glucose; HC, hip circumference; HDL, high-density lipoprotein; HOMA-IR, homeostatic model assessment of insulin resistance; HOMA-β, homeostasis model assessment of β-cell dysfunction; hs-CRP, high-sensitive C-reactive protein; LDL, low-density lipoprotein; NC, neck circumference; NHANES, National Health and Nutrition Examination Survey; NPREE, nonprotein resting energy expenditure; NPRER, nonprotein respiratory exchange ratio; RCT, randomized controlled trial; REE, resting energy expenditure; RQ, respiratory quotient; SBP, systolic blood pressure; SFT, skinfold thickness; TC, total cholesterol; TG, triglyceride; Vo_2peak_, peak oxygen uptake; WC, waist circumference; WHR, waist to hip ratio.

## THE ROLE OF CITRUS METABOLITES IN ENERGY METABOLISM

### Dietary fiber (citrus peel)

Citrus peel, which contains flavonoids, essential oils, and vitamins, is also a rich source of dietary fiber.^60^ According to their solubility, dietary fibers are divided into digestible dietary fiber (pectin) and nondigestible dietary fibers (cellulose, hemicellulose, and lignin).^61^ Although digestible dietary fibers have beneficial effects on blood sugar and cholesterol, nondigestible dietary fibers are essential for colon health.^62^ Citrus peels contain >60% dietary fiber, the majority of which is nondigestible dietary fiber.^63^ Although the history of the use of citrus peels dates back to the 10th century, the explanation of the activities of the components in the peel dates back to more recent times.^64^ It has been reported that boosting dietary fiber intake elevates the production of short-chain fatty acids in the intestine, supporting energy expenditure and aiding weight loss.^65^ In the study conducted by Chambers et al,^66^ the intake of propionate, the end product of dietary fiber fermentation, increased resting energy expenditure in 18 people aged 18–65 years. In another study carried out with rats fed a high-fat diet (HFD), extracts of orange, lemon, grapefruit, and tangerine peels were administered separately and in equal amounts in a combination to examine the effect on weight loss. Decreased appetite was reported in all groups, due to the satiety in the stomach caused by the pectin found in the citrus peels. Although the most beneficial effect in weight loss was observed in the group given the combination of all extracts, this effect was reported to be because of the stimulation of β-3 cell receptors and increased thermogenesis.^67^  *C. sunki* peel extract^68^ and *C. ichangensis* peel extract^69^ were slowed weight gain through β-oxidation and lipolysis in mice fed a HFD.

### Citrus flavonoids

Among the citrus flavonoids,^70^ some of which are responsible for the bitter taste of citrus, the most important group is flavanones (namely, hesperidin, hesperetin, naringenin, naringin, narirutin, eriocitrin, neohesperidin, dydimin, poncirin, and neoeriocitrin), flavones (tangeretin and nobiletin), and flavonols (quercetin, kaempferol).^71^^,^^72^ Additionally, citrus peel polymethoxyflavones (PMFs) are considered an important source of flavanone.^73^ However, the amount and subgroups of these flavonoids vary according to both citrus type and citrus tissue.^72^^,^^74^ Moreover, the type of fruit juice–processing technology and processing processes can also significantly reduce the flavonoid content.^75^^,^^76^ For this reason, the results of in vivo studies using citrus should be evaluated considering this difference in terms of citrus fruit,^77^ fruit juice,^78^ fruit peel,^79^ vitamins, and flavonoids.

#### Hesperidin

**Figure 1:**
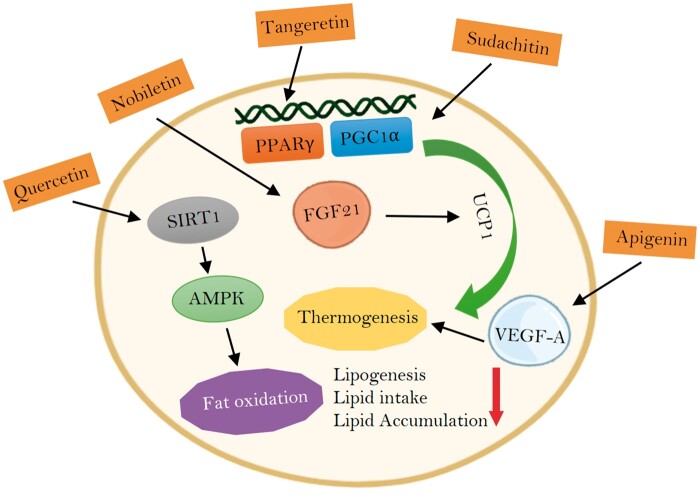
**Metabolic effects exhibited by various pathways of citrus fruit flavonoids.** AMPK, AMP-activated protein kinase; FGF-21, fibroblast growth factor 21; PGC, peroxisome proliferator-activated receptor-gamma coactivator-1α; PPARγ, peroxisome proliferator-activated receptor-γ; SIRT1, sirtuin 1; UCP1, uncoupling protein 1; VEGF-A, vascular endothelial growth factor A.

Citrus flavonoids can mediate energy expenditure in a variety of ways (Figure 1). Nishikawa et al^80^ showed that α-monoglucosyl hesperidin, the synthetic form of hesperidin with higher bioavailability and solubility, induced brown-fat adipocyte formation in mice and increased thermogenesis via uncoupled protein 1 (ucp-1) in white adipose tissue (WAT). Similarly, Shen et al^81^ reported that oral administration of 4G-α-glucopyranosyl hesperidin, a form of hesperidin with a greater absorption and solubility level, strengthened interscapular brown adipose tissue (BAT) sympathetic neuronal activity in rats, and brown fat caused by increased body temperature suggested increased thermogenesis in the tissue. Ohara et al,^82^ on the other hand, found that the treatment of glycosyl hesperidin and caffeine together in mice increased the expression of adipose tissue mass and liver lipogenic gene messenger RNAs (mRNAs), but the treatment alone did not show a significant effect. There is a limited number of human studies on this subject. According to the results of a randomized, placebo-controlled study of 40 amateur cyclists who were given 2*S*-hesperidin supplements for 8 weeks, a decrease in total fat mass and an increase in muscle mass were reported in the hesperidin group.^19^ In another randomized, placebo-controlled study, conducted by Yoshitomi et al^83^ with 60 healthy Japanese individuals aged 30–75 years who were given combined green tea and α-glucosyl hesperidin, body weight and visceral fat area were decreased in the entire intervention group. Individuals younger than 50 years in subgroup analyses had significant reductions in body weight, body fat percentage, visceral fat, and total abdominal fat areas.^83^

#### Naringenin

Naringenin, another citrus flavanone, increased thermogenesis through ucp1, and pgc-1α upregulation, and thus lipid accumulation in BAT, in a murine study conducted by Bae et al.^84^ Zhang et al,^85^ on the other hand, showed that treatment of naringenin in mice fed an HFD was effective in preventing HFD-induced obesity and in increasing energy expenditure by activating thermogenesis and beiging through gut microbe–short-chain fatty acid–host interactions. In other animal studies, it has been shown that naringenin supplementation reduces intra-abdominal and subcutaneous adiposity,^86^ increases the expression of fat oxidation genes and β-hydroxybutyrate concentration in the liver,^87^ and contributes to energy expenditure by increasing mitochondrial biogenesis.^88^ Rebello et al^89^ showed that naringenin treatment of human white adipocyte cultures and abdominal subcutaneous adipose tissue promoted beiging by increasing the expression of key enzymes related to thermogenesis and fat oxidation. In vivo human studies involving naringenin supplementation appear promising in reducing weight, BMI, waist circumference, and visceral fat levels; however, more studies are needed.^90–92^

#### Eriocitrin

Eriocitrin flavanone, which is abundant in lemons, increased the mRNA expression of genes associated with thermogenesis and energy expenditure in mice to which it was administrated for 16 weeks and decreased lipogenesis and lipid uptake–related gene expression in WAT.^93^ The results of a randomized controlled trial using nutraceutical supplementation of this flavanone, which has been the subject of limited studies, show that eriocitrin is not effective in terms of weight, waist circumference, and BMI change.^94^

#### Tangeretin and nobiletin

Supplementation of tangeretin, a citrus flavone, to the diet of HFD-fed mice improved obesity by increasing thermogenic gene expression and reducing intestinal dysbiosis.^95^ In the in vitro study of Kihara-Negishi et al,^96^ another flavone, nobiletin, increased UCP-1 expression in HB2 brown adipocyte cell lines and mRNA expression of brown adipokines neuregulin-4 and fibroblast growth factor 21, and also has been suggested to activate BAT through β-adrenergic stimulation by increasing the secretion of fibroblast growth factor-21. Another in vitro study demonstrated the antiadipogenic effect of nobiletin in 3T3-L1 cells (a preadipocyte cell line) by modulating the peroxisome proliferator–activated receptor-γ (PPARγ) and AMP-activated protein kinase (AMPK) pathway.^97^ In contrast, an animal study suggested that the metabolic effects of nobiletin are independent of AMPK activation.^98^ Kou et al^99^ showed that nobiletin supplementation activates thermogenesis in BAT and WAT through its positive effects on gut microbiota in mice fed an HFD.

#### Apigenin

Another flavone found in citrus is apigenin.^72^ Apigenin can protect against inflammation and support beiging by activating the COX2/PGE2 axis in human adipocytes.^100^ In 3T3-L1 cells, C/EBPβ (a liver-enriched activating protein), a transcription factor involved in adipocyte differentiation) upregulates the expression of C/EBPβ inhibitors.^101^^,^^102^ Another study showed that WAT browning occurs by triggering the expression of vascular endothelial growth factor A.^103^ Sun and Qu,^104^ on the other hand, suggested that 12-week apigenin treatment in mice can reduce body weight by increasing energy expenditure without a change in energy intake. The number of studies on the effect of luteolin on energy expenditure is limited; it is thought to contribute by increasing browning and thermogenesis via AMPK/PGC1α.^105^

#### Quercetin, rutin, and kaempferol

There are in vivo human and animal studies in which the effect of quercetin, a citrus flavonol, on energy metabolism was examined. Some studies show that quercetin contributes to browning by increasing the expression of thermogenesis genes without increasing energy expenditure,^106–108^ whereas others show that it promotes lipophagy and prevents adiposity through the activation of AMPK signaling.^109^^,^^110^ Clinical studies have shown no significant effect of quercetin supplementation on REE,^78^ basal metabolic rate,^79^ total energy expenditure,^79^ body weight,^111–113^ body fat mass,^111^^,^^112^ lean body mass,^111^^,^^112^ and WHR.^111^^,^^113^ The glycosyl derivatives of quercetin, rutin, and isoquercetin are also found in citrus. Rutin can increase energy expenditure by increasing the expression of ucp-1 and other thermogenic genes^20^^,^^114^ and upregulating the AMPK pathway.^115^ In addition, in a clinical study using whey protein powder with added isoquercitrin, no significant effect was observed on total body mass, lean mass, and fat mass.^116^ Kaempferol, another flavonol, upregulated the cAMP-responsive gene for type 2 iodothyronine deiodinase by increasing the intracellular energy expenditure.^117^

#### Other flavonoids

Another component that contains more than 1 methoxy group on the flavone skeleton, which is abundant in citrus peels, are PMFs.^118^ Although there are more than 80 types of PMFs, which are specific to citrus fruits, the main ones found in citrus peels are tangeretin, nobiletin, sudachitin, and sinensetin.^119^ Administration of PMF-containing extracts obtained from citrus peels to mice fed an HFD reduced weight gain.^79^,^120–124^ Dietary supplements containing PMFs attenuate adipogenic differentiation by downregulating adipogenic factors such as PPARγ and upregulating AMPK in 3T3 L1 cells,^125^ whereas citrus peel extracts reduced mouse body weight and adipose tissue weight through microbiota or AMPK activation.^120–122^ Sudachitin extract, thought to result from sudachitin polyphenol found in the peel of *C. sudachi*, increases energy expenditure by increasing SIRT1, PGC1-α, and UCP-1 gene expression in skeletal muscle.^126^ Extracts containing the PMFs also reduced weight gain.^127–130^ To prevent obesity, PMFs play an important role in preventing hypertrophy by inhibiting lipid accumulation and apoptosis in 3T3-L1 cells.^131^^,^^132^ More in vivo and in vitro studies, especially clinical studies, are needed on the effect of citrus flavonoids on energy metabolism.

### Citrus carotenoids and terpenes

Carotenoids are fat-soluble pigments responsible for the red, orange, and yellow coloration of citrus. Carotenoids, which are highly sensitive to enzymatic, chemical, and oxidative reactions, may cause different responses even if the dietary intake of individuals is the same. Therefore, when investigating their therapeutic effects, the biology, activity, and metabolism of carotenoids should be well investigated.^133^^,^^134^ As with flavonoids, carotenoids can be found in citrus in varying amounts depending on environmental conditions or species. β,β-Xanthophylls (primarily β-cryptoxanthin, zeaxanthin/lutein, and violaxanthin) constitute 90% of the carotenoid content of citrus, which contains approximately 115 different carotenoids.^135^ Terpenes, on the other hand, are the main components of essential oils, among the main ones in citrus are limonene, α-terpineol, β-pinene, carene, myrcene, and linalool, that give unique taste and odor to plants.^136^^,^^137^

β-Cryptoxanthin, occurs mainly in tangerine and sweet orange, and has been reported to have a higher effect on fat reduction and protection against oxidative stress at low doses compared with lycopene and β-carotene.^138^ Additionally, studies have reported that dietary consumption of tangerine increases serum β-cryptoxanthin levels in humans.^139^^,^^140^ Treatment of HFD-fed mice with β-cryptoxanthin, which is stored in adipose tissue after absorption, increased ucp-1 expression via the retinoic acid receptor pathway,^141^ increasing energy expenditure and reducing lipid accumulation. Xie et al^142^ also showed that zeaxanthin, another carotenoid, activates the β3-adrenergic receptor in HFD-fed mice, increasing the expression of prdm16, pgc-1α, and ucp-1; and WAT thermogenesis. The results of the meta-analysis conducted by Yao et al,^143^ including studies evaluating serum levels of β-cryptoxanthin and lutein/zeaxanthin,^144–146^ found a significant relationship between carotenoids and reduction of body weight, BMI, and waist circumference in overweight and obese individuals.

Limonene, 1 of the citrus terpenes, increased the expression of PRDM16 and UCP-1, and also modulated C/EBPβ in 3 T-L1 cells in the in vitro study of Lone and Yun.^147^ It was thought that it could induce beiging, as well.^147^

There are not enough in vitro and in vivo studies investigating the mechanism of action of citrus carotenoids and terpenes on energy expenditure. More studies are needed to elucidate this area.

### Citrus alkaloids

Alkaloids of citrus metabolites are octopamine, synephrine (*p*-synephrine, *m*-synephrine, *o*-synephrine, and methylsynephrine), tyramine, *N*-methyltyramine, and hordenine.^148^  *p*-Synephrine, which is found as a primary alkaloid in many citrus fruits, has a key role in increasing energy expenditure and controlling body weight.^6^  *p*-Synephrine, the most common source of which is *C. aurantium* (bitter orange),^149^ is a phenylethylamine derivative with a hydroxyl group on the benzene ring.^26^  *C. aurantium* extract is standardized to contain 6%–10% *p*-synephrine.^150^ Ephedrine, popular for body weight loss because of its effect causing increased thermogenesis and decreased appetite, was banned for causing hypertension, heart disease, and stroke.^151^ Since then, the use of synephrine, which is like ephedrine in its structure and mechanism of action, has attracted attention.^152^

The amount of synephrine is higher in unripe citrus fruits and decreases as they mature. The amount of synephrine in *C. deliciosa* was found to be higher than in other citrus fruits.^6^^,^^24^ The French Agency for Food, Environmental and Occupational Health and Safety reported that the level of synephrine intake from food supplements should be <20 mg/d and should not be taken in combination with caffeine.^119^

*p*-Synephrine affects the resting metabolic rate by binding and stimulating β-3 adrenoceptors.^153^ It has been reported that activation of β-3 adrenoreceptors increases lipolysis in adipose tissue and decreases weight gain.^154^  *p*-Synephrine also reduces aP2 expression and lipid accumulation in 3T3-L1 cells.^155^ It has been reported that β-synephrine causes significant increases in resting metabolic rate in humans, and its use for up to 12 weeks can result in moderate weight loss.^156^ Stohs et al^25^ examined the effect of the consumption of *p*-synephrine alone or in combination with some flavonoids on the resting metabolic rate, and they reported that 50 mg of *p*-synephrine supplementation alone increased the resting metabolic rate by 6.9% compared with placebo. Jung et al^157^ reported that 20 mg of *p*-synephrine supplementation before training by 25 participants increased the respiratory quotient significantly compared with the placebo.

Gutiérrez‐Hellín and Del Coso^158^ showed that 3 mg/kg *p*-synephrine did not affect resting energy expenditure and fat oxidation compared with placebo. However, it can increase fat oxidation without affecting energy expenditure during exercise.^159–161^ Ratamess et al^162^ examined the effects of β-synephrine supplementation alone and in combination with caffeine on resistance exercise performance in 12 healthy men. The use of 100 mg of *p*-synephrine in the study provided significant increases in the number of repetitions compared with the placebo and control groups. Gougeon et al^163^ investigated whether alkaloids increase the thermic effect of food. They reported that the thermic effect was 20% lower in women than men after eating only, and the intake of capsule alkaloids (namely, synephrine, hordenine, octopamine, tyramine, and *N*-methyltyramine) with a meal increased the thermic effect by 29% only in women. Capsule use significantly increased the respiratory quotient in both sexes, whereas no change was observed in blood pressure. Hoffman et al^164^ found that a capsule containing 20 mg of methylsynephrine and methylhordenine together with caffeine tetradecylthioacetic acid, yerba mate extract, and methylphenylethylamine significantly increased the average 3-hour energy expenditure. Kaats et al^165^ tested the safety of synephrine with 67 individuals who consumed 50 mg of *p*-synephrine alone or together with citrus flavonoids (naringin and hesperidin) for 60 days. At the end of the study, there was no significant change in the systolic or diastolic blood pressures and blood findings of the groups.

## EFFECTS OF CITRUS FRUIT CONSUMPTION ON CLINICAL OUTCOMES

In vitro and in vivo animal studies mentioned in the previous section discussed in detail that citrus bioactive components have an effect on energy expenditure through various pathways such as thermogenesis, beiging, or fat oxidation (Figure 1). Although these studies generally reported positive effects, direct consumption of a citrus component and consumption as a fruit juice or citrus fruit extract may not have the same effects on the organism, especially considering the variation of flavonoid amounts in the citrus type and processing duration.^74–76^ Therefore, it would not be correct to generalize the positive effects of a citrus component consumed as citrus fruit or fruit juice, in terms of preclinical studies to clinical studies. On the other hand, it is reasonable to base positive effects seen in clinical studies on theories from preclinical studies. In this section, we discuss the effects of consuming citrus fruits as fresh, juice, or extract in clinical studies on anthropometric and biochemical outcomes in humans.

It is widely accepted that if environmental factors such as dietary energy intake and physical activity change, the composition of the human body will change. Negative energy balance is associated with a significant reduction in body weight and visceral adipose tissue.^166^^,^^167^ The studies included in this review reported the effects of citrus consumption on anthropometric measures such as body weight, BMI, body fat, and waist, hip, and neck circumference. Of the 28 studies evaluating body weight and BMI, only 8 reported that citrus was effective in reducing body weight and BMI. Body fat mass was decreased in 4 of 12 studies, and body fat mass and body weight changes were correlated. Circumference measurements (waist, hip, neck) did not change significantly in most studies. Studies with positive results did not show a weighted distribution to any citrus species. However, the remarkable point is that the extract form is reported to be more effective than consuming citrus as fresh or fruit juice. Studies conducted using any of these consumption forms by humans and the observed effects are summarized in Table 2.^27–59^

Citruses can inhibit lipid accumulation by activating AMPK signaling and the PPARγ/AMPK pathway^97^ or increasing thermogenesis through ucp-1 expression.^141^ BAT has an active role in energy expenditure through thermogenesis.^168^ Increasing the activity of BAT can boost energy expenditure^169^ and may also lower blood lipid levels.^170^ A dysfunction in the AMPK pathway is linked to fat accumulation and high blood lipid levels.^171^ Based on these findings, the effect of citrus fruits on thermogenesis and their impact on triglycerides (TGs), total cholesterol (TC), and low-density lipoprotein (LDL) and high-density lipoprotein (HDL) levels, which are biochemical markers of lipid metabolism, is examined.

Consumption of 750 mL/d orange juice for 8 weeks significantly reduced TC,^35^ LDL,^35^ and HDL^51^ levels in adults. Consumption of 500 mL/d orange juice for the same duration in women also significantly reduced TC and LDL levels.^34^ Additionally, it significantly increased the HDL level in women and significantly decreased the TG levels in men.^34^ Consumption of 500 mL of orange juice for 12 weeks significantly decreased TG levels.^48^ Combining a low-energy diet with the same amount of orange juice consumption for the same duration significantly decreased the LDL^44^ level, whereas combining exercise decreased TC and LDL levels, and increased HDL and TG^42^ levels. Reducing the amount of orange juice to 300 mL/d for 60 days significantly decreased^47^ TG, TC, and LDL levels. As a result of a dose of 250 mL/d orange juice, LDL, HDL, and TC levels did not change.^33^^,^^43^ As seen in the studies, although orange juice consumption decreases TG, TC, and LDL levels and increases HDL levels in 2–3 months, these results are not consistent. This inconsistency may be due to the amount of orange juice consumption, duration, flavonoid content, and health status of the participants. When looking at the effects of different types of citruses, citrus lime consumption for 8 weeks significantly reduced LDL, TC, and TG levels.^56^ Consumption of half a grapefruit^55^ and lemon peel powder^57^ significantly reduced TC and LDL levels. Citruses also show activity in different biochemical findings. Orange juice consumption significantly decreased fasting glucose levels,^51^^,^^56^ homeostatic model assessment of insulin resistance values,^47^^,^^51^ and insulin^47^ and high-sensitivity C-reactive protein^172^ levels.

## CONCLUSIONS

Studies including citruses have explained the effect on energy metabolism and lipid oxidation of citrus components: dietary fiber, flavonoids, alkaloids, terpenes, and carotenoids. Citruses increase energy expenditure by increasing thermogenesis through stimulation of β-3 cell receptors and increasing mitochondrial biogenesis. In addition, BAT browning and increased thermogenesis are observed through AMPK/PGC1α or triggering vascular endothelial growth factor A expression. Similarly, energy expenditure is increased by increasing pgc1-α, sırt1, and ucp-1 gene expression and activating the β3-adrenergic receptor. In in vitro studies, citrus fruits had antiadipogenic effects by activating the AMPK signal and the PPARγ/AMPK pathway and inhibiting lipid accumulation in 3T3-L1 cells. Additionally, lipid accumulation is inhibited by the expression of UCP-1. Citrus increases mRNA expression of genes associated with energy expenditure and decreasing lipogenesis-related gene expression in WAT. It also upregulates the cAMP-responsive gene for type 2 iodothyronine deiodinase, increasing intracellular energy expenditure.

Even though in vivo animal studies and in vitro studies have reported the positive effects of citrus components, it is not possible to clearly say that citrus consumption has the same effect in humans when looking at clinical studies. Studies reporting the significant effects of citrus fruits on anthropometric and biochemical findings are limited. In addition, although the mechanisms by which citrus fruits increase energy expenditure and result in weight loss have been shown, there are limited clinical studies evaluating energy expenditure. This indicates the dose–time interaction that was reported to have efficacy in vivo animal studies would not have the same effect as adding citrus fruits to the human diet. This does not mean that citruses are not beneficial for human health and metabolism, but it does indicate that it is premature to recommend adding them to the diet in terms of promising weight loss.

This comprehensive review summarizes how citrus metabolites affect energy metabolism in different in vitro and in vivo animal studies and the results of different citrus varieties in human studies. More studies are needed on the effect of citrus on energy expenditure.
